# *In vivo* imaging of Zika virus reveals dynamics of viral invasion in immune-sheltered tissues and vertical propagation during pregnancy

**DOI:** 10.7150/thno.43177

**Published:** 2020-05-16

**Authors:** Ting Wang, Penghui Li, Yuan Zhang, Yan Liu, Zhongyuan Tan, Jianhong Sun, Xianliang Ke, Yuanjiu Miao, Dan Luo, Qinxue Hu, Fuqiang Xu, Hanzhong Wang, Zhenhua Zheng

**Affiliations:** 1 CAS Key Laboratory of Special Pathogens and Biosafety, Center for Emerging Infectious Diseases, Wuhan Institute of Virology, Chinese Academy of Sciences, Wuhan 430071, China; 2 Center for Brain Science, State Key Laboratory of Magnetic Resonance and Atomic and Molecular Physics, Key Laboratory of Magnetic Resonance in Biological Systems, Wuhan Center for Magnetic Resonance, Wuhan Institute of Physics and Mathematics, Chinese Academy of Sciences, Wuhan 430071, China; 3 College of Life Sciences and Food Engineering, Hebei University of Engineering, Handan 056038, China; 4 State Key Laboratory of Virology, Wuhan Institute of Virology, Chinese Academy of Sciences, Wuhan 430071, China; 5 Institute for Infection and Immunity, St George's, University of London, London, SW17 0RE, UK

**Keywords:** Zika virus, Bioluminescence imaging, Viscera dissemination, Tissue localization, Vertical transmission, Pooled immune sera

## Abstract

**Rationale**: Zika virus (ZIKV) is a pathogenic virus known to cause a wide range of congenital abnormalities, including microcephaly, Guillain-Barre syndrome, meningoencephalitis, and other neurological complications, in humans. This study investigated the noninvasive detection of ZIKV infection *in vivo*, which is necessary for elucidating the virus's mechanisms of viral replication and pathogenesis, as well as to accelerate the development of anti-ZIKV therapeutic strategies.

**Methods**: In this study, a recombinant ZIKV harbouring Nluc gene (ZIKV-Nluc) was designed, recovered, and purified. The levels of bioluminescence were directly correlated with viral loads *in vitro* and *in vivo*. The dynamics of ZIKV infection in A129 (interferon (IFN)-α/β receptor deficient), AG6 (IFN-α/β and IFN-γ receptor deficient), and C57BL/6 mice were characterized. Pregnant dams were infected with ZIKV-Nluc at E10 via intra footpad injection. Then, the pooled immune sera (anti-ZIKV neutralizing antibodies) #22-1 in ZIKV-Nluc virus-infected mice were visualized.

**Results**: ZIKV-Nluc showed a high genetic stability and replicated well in cells with similar properties to the wild-type ZIKV (ZIKVwt). Striking bioluminescence signals were consistently observed in animal organs, including spleen, intestine, testis, uterus/ovary, and kidney. The ileocecal junction was found to be the crucial visceral target. Infection of pregnant dams with ZIKV-Nluc showed that ZIKV was capable of crossing the maternal-fetal barrier to infect the fetuses via vertical transmission. Furthermore, it was visualized that treatment with the pooled immune sera was found to greatly restrict the spread of the ZIKV-Nluc virus in mice.

**Conclusions**: This study is the first to report the real-time noninvasive tracking of the progression of ZIKV invading immune-sheltered tissues and propagating vertically during pregnancy. The results demonstrate that ZIKV-Nluc represents a powerful tool for the study of the replication, dissemination, pathogenesis, and treatment of ZIKV *in vitro* and *in vivo*.

## Introduction

Zika virus (ZIKV) is a mosquito-borne virus that belongs to the *Flaviviridae* family 1. ZIKV was first isolated in 1947 from a febrile rhesus macaque in the Zika forest of Uganda 2. Only 14 cases of sporadic infection with mild symptoms were reported in the following 60 years 3-5. After several epidemics in Yap Island in 2007 and in French Polynesia and other Pacific islands in 2013-2014, ZIKV colonised the Americas in February 2015, causing 707,133 cases of autochthonous infection within 48 countries and territories by late 2016 3, 6, 7. During these large epidemics in the Americas, ZIKV infection resulted in severe pathological complications, notably microcephaly in newborns and Guillain-Barré syndrome (GBS) in adults 4, 6, 8. The virus is mainly transmitted by the bite of *Aedes* mosquitoes, but direct, interhuman transmission through sexual or vertical route has also been confirmed, setting ZIKV apart from most other flaviviruses 9. To date, there is no licensed vaccine or antiviral therapy available for the treatment of ZIKV infection. The efficient transmission of this virus combined with deficient antiviral strategies has exacerbated public panic over ZIKV 10.

The mechanisms for the dissemination and pathogenesis of ZIKV in developing fetuses, pregnant mothers, and adults remain largely unknown. ZIKV is likely to invade a unique set of immune-sheltered tissues, including the brain, testis, and placenta. Several ZIKV animal infection models have been previously established 11 to quantify viral genomes and antigens, which have provided useful information about both the viral and host factors that determine replication and pathogenesis 12-14. However, it has not been possible to monitor the real-time patterns of ZIKV infection through these methods 13. The collection of tissues and organs to evaluate ZIKV infection requires the euthanasia of the animals, and important tissues or organs may be missed if samples are not taken adequately 13, 14.

Bioluminescence imaging is a sensitive and non-invasive technology that allows for the visualization of viral dynamics in real time 15, 16. This strategy measures the light generated by luciferase-catalysed oxidation reactions, an indicator of the extent of infected tissues, by using a charge-coupled device (CCD) camera 17. Bioluminescence imaging measures the spatial and temporal progression of both primary infection and reinfection in the same animal model, which is able to not only reduce the inter-animal variability and animal suffering, but also improve the accuracy, stability, and reproducibility of the results 18, 19. Bioluminescence imaging has been widely utilised in the study of viruses, including influenza virus, enterovirus 71, herpes simplex virus, respiratory syncytial virus, dengue virus, Japanese encephalitis virus, monkeypox virus, and hepatitis C virus 15-17, 19-23. Recently, bioluminescence imaging assays of flaviviruses infection in mice have been implemented using recombinant viruses harbouring the firefly luciferase (Fluc) or Renilla luciferase (Rluc) gene 19, 20, 24. Compared with Fluc and Rluc, the very small nanoluciferase (Nluc) (19-kDa) produces 150-fold more light 17, 25, and shows a greater potential for bioluminescence imaging 26. To date, there have been no successful attempts at the non-invasive detection of ZIKV infection *in vivo*, which is warranted for characterizing the mechanisms of the replication and pathogenesis of the virus, as well as to improve the preclinical evaluation of vaccines, antiviral drugs, or therapeutic antibodies for ZIKV.

In this study, a recombinant ZIKV harbouring the Nluc gene (ZIKV-Nluc) was designed, recovered, and purified. This recombinant virus showed a high genetic stability and replicated well in cells with similar properties to the wild-type ZIKV (ZIKVwt). The levels of bioluminescence were found to be directly correlated with viral loads *in vitro* and* in vivo*. The dynamics of ZIKV infection in A129 (interferon (IFN)-α/β receptor deficient), AG6 (IFN-α/β and IFN-γ receptor deficient), and C57BL/6 mice were well characterized. To our knowledge, this is the first real-time non-invasive tracking of the progression of ZIKV invading immune-sheltered tissues, as well as its vertical propagating, during pregnancy. The results presented in this study demonstrate that ZIKV-Nluc is a powerful tool for use in the study of the replication, dissemination, and pathogenesis of ZIKV *in vitro* and* in vivo*.

## Results

### Generation and characterization of a stable reporter ZIKV

**Figure 1:**
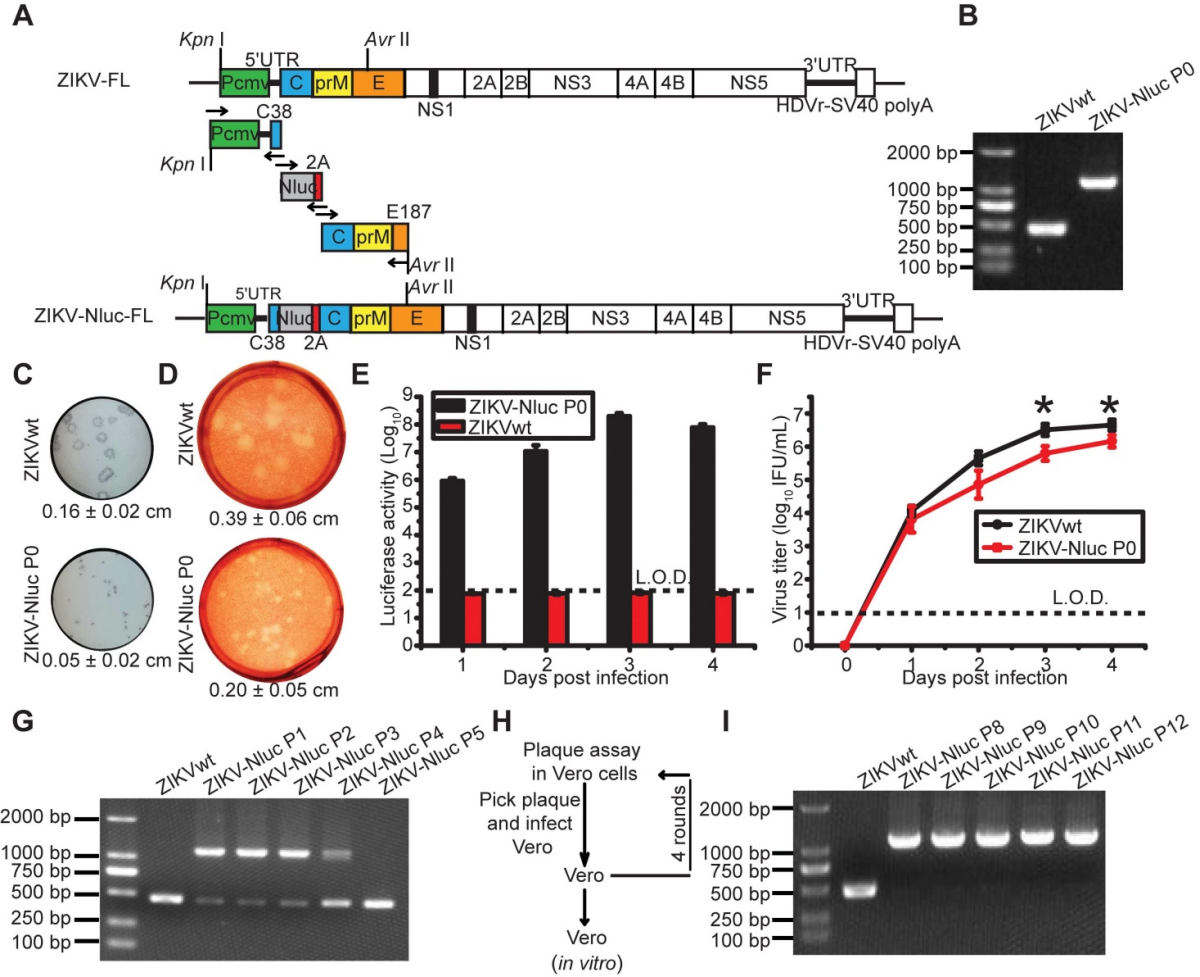
** Generation and characterization of ZIKV-Nluc.** (A) Strategy for constructing the full-length cDNA clone of ZIKV-Nluc. The monomeric Nluc gene flanked by the N-terminal 38 amino acids of C protein (C38) and a FMDV2A (F2A) sequence was inserted between 5'UTR and the C gene. (B) The stability of the P0 ZIKV-Nluc virus. Viral RNAs were extracted from the supernatants, and RT-PCR was performed with a pair of primers surrounding the Nluc-2A fragment. (C) The plaque morphology of the P0 ZIKV-Nluc virus in Vero cells, visualized by immunostaining following incubation for 4 days. (D) The plaque morphology of the P0 ZIKV-Nluc virus in Vero cells, visualized using 0.33% neutral red following incubation for 5 days. (C, D) The average sizes of viral plaques (mean ± standard deviation) were quantified by counting all of the intact plaques. (E) Nluc activities of the infected Vero lysates by P0 ZIKV-Nluc virus at different times post infection at low multiplicity of infection (MOI) of 0.01. (F) Growth curves of P0 ZIKV-Nluc virus determined by an immunostaining plaque assay at an MOI of 0.01. (G) ZIKV-Nluc stability during virus passaging. Total RNA was extracted from the cells infected by each passaged virus, and RT-PCR was performed with a pair of primers surrounding the Nluc-2A fragment. (H) Schematic of plaque purification. (I) ZIKV-Nluc stability following plaque purification. Data represent the mean ± SD analysed by Student's t-test (two tailed) (*, P < 0.05; **, P < 0.01; ***, P < 0.001).

To generate a bioluminescent ZIKV reporter virus, a Nluc gene was engineered into a full-length infectious cDNA clone of an Asian-lineage Zika virus, SZ-WIV01 10. As shown in Figure 1A, the monomeric Nluc gene flanked by the N-terminal 38 amino acids of C protein (C38) and a FMDV2A (F2A) sequence was inserted between 5'UTR and the N-terminus of open reading frame (ORF). The C38 sequence was required for maintaining genome cyclization and for viral RNA replication, and the F2A sequence was placed downstream of the Nluc gene to ensure that the Nluc protein was properly processed 19, 27.

P0 ZIKV-Nluc virus was rescued through the transfection of Vero cells with the full-length ZIKV-Nluc cDNA clone (ZIKV-Nluc-FL). P0 ZIKV-Nluc virus harbouring the intact genome, without the loss of Nluc, was confirmed by RT-PCR assay by amplifying the fragment from 5'UTR to the C gene (Figure 1B). Compared with ZIKVwt, the P0 ZIKV-Nluc virus produced smaller plaques, which were visualized by immunostaining (P < 0.001) (Figure 1C) and 0.33% neutral red (P < 0.001) (Figure 1D), demonstrating that the insertion of the Nluc gene attenuated the virus in cell culture. The infection of Vero cells with P0 virus resulted in the robust production of luciferase activity (Figure 1E), despite the P0 virus having lower infectious titres, at 3 and 4 days post infection (dpi) (P < 0.05), and exhibiting a lower replication efficiency than ZIKVwt (Figure 1F).

The genetic stability of ZIKV-Nluc is prerequisite for its use. To test the stability of the ZIKV-Nluc virus, the P0 ZIKV-Nluc virus was passaged in Vero cells for five rounds. After each passage (P1 to P5), the viruses were examined for the Nluc gene. The RT-PCR results indicated that the Nluc gene began to be lost in the P1 viral stock (Figure 1G). To improve the stability of ZIKV-Nluc, the P0 ZIKV-Nluc virus was purified for four rounds in Vero cells using a double plaque assay (Figure 1H). The resulting P8 ZIKV-Nluc virus was re-passaged five times in Vero cells, the passages of which did not result in any apparent loss of NLuc gene, indicating that the genome of ZIKV-Nluc was stable for at least five life cycles (Figure 1I). Sequence analysis of the entire genome of P8 ZIKV-Nluc virus revealed no nucleotide substitution (Fasta data-S1 in Supplementary material).

**Figure 2:**
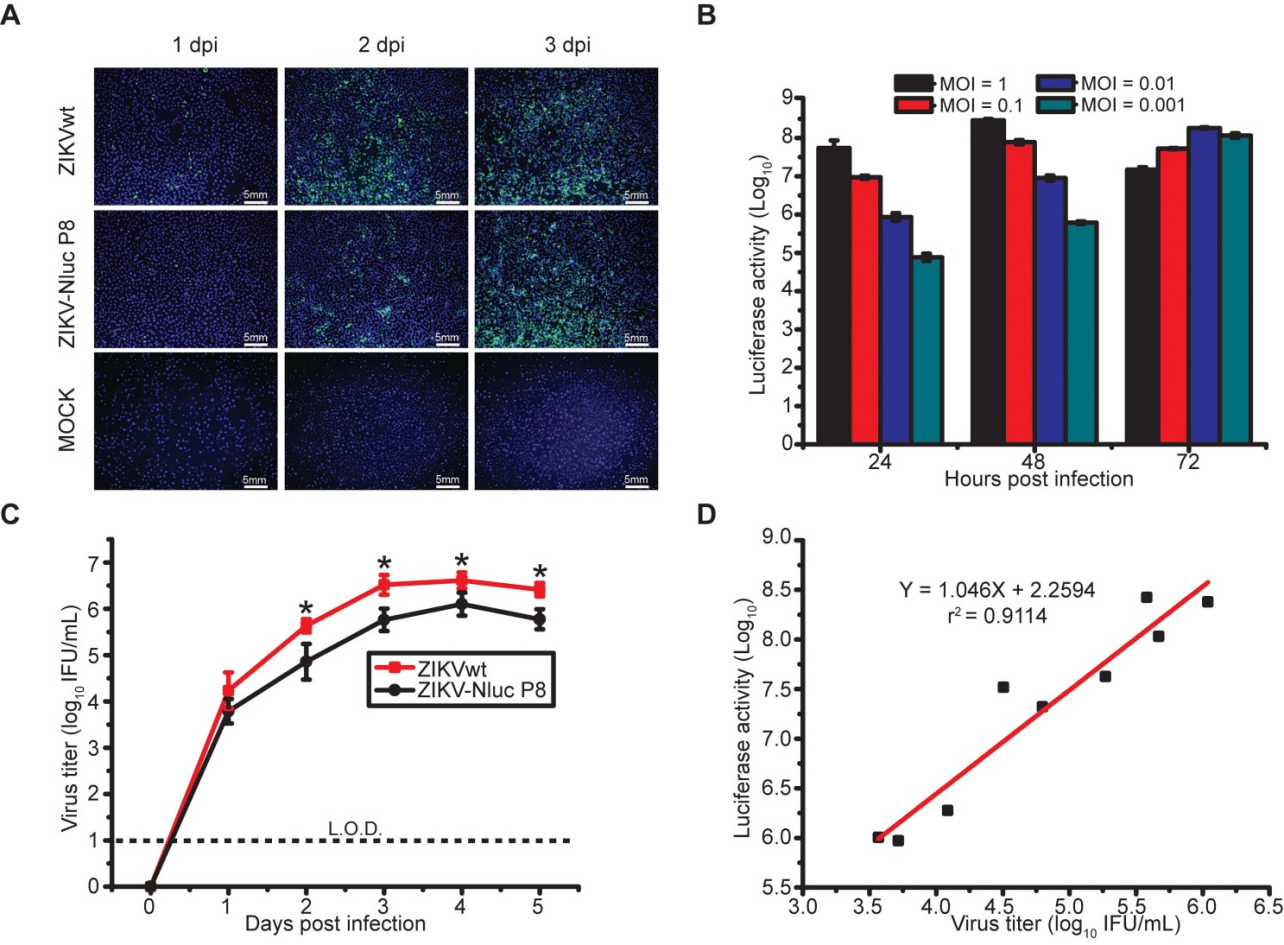
** Replication of ZIKV-Nluc in cell culture.** (A) IFA of E protein expression in Vero cells infected with the purified ZIKV-Nluc virus at an MOI of 0.05. (B) Nluc activities of infected Vero lysates by the purified ZIKV-Nluc virus at different times post infection at an MOI of 0.001, 0.01, 0.1, and 1, respectively. (C) Growth curves of the purified ZIKV-Nluc virus determined by immunostaining plaque assay at an MOI of 0.01. (D) Linear correlation between viral titres and Nluc signal values of the purified ZIKV-Nluc virus *in vitro*. Data represent the mean ± SD analysed by Student's t-test (two tailed) (*, P < 0.05; **, P < 0.01; ***, P < 0.001).

To further validate the ZIKV-Nluc virus (P8), viral protein synthesis was examined in Vero cells. As measured by IFA assays, the amount of E-positive cells infected with ZIKV-Nluc virus increased with the time of infection, although the percentage of positive cells was less than that of ZIKVwt before the peak at 3 dpi (Figure 2A). Next, we determined the kinetics of the luciferase activities in Vero cells infected with different MOIs (0.001, 0.01, 0.1 or 1) of ZIKV-Nluc virus. As shown in Figure 2B, the growth of the Nluc signal showed a good correlation with the MOI in the ZIKV-Nluc virus (P8)-infected Vero cells. In a parallel experiment of double plaque assay, P8 ZIKV-Nluc virus showed low infectious titres at 2, 3, 4, and 5 dpi (P < 0.05). However, the growth pattern for the bioluminescent virus was similar to that for ZIKVwt (Figure 2C). In addition, the MOI of 0.01 was selected and applied in subsequent correlation analyses of viral titres and Nluc activities. We demonstrated that there was an excellent linear correlation (r^2^ = 0.9114) between the Nluc signal values and the viral titres of P8 ZIKV-Nluc virus (Figure 2D). Collectively, the P8 ZIKV-Nluc virus showed a superior genetic stability and produced levels of luciferase activity that accurately reflected the replication of the virus* in vitro*. Therefore, this virus was used in further experiments.

### Pathogenicity of ZIKV-Nluc in A129, AG6, and C57BL/6 mice

**Figure 3:**
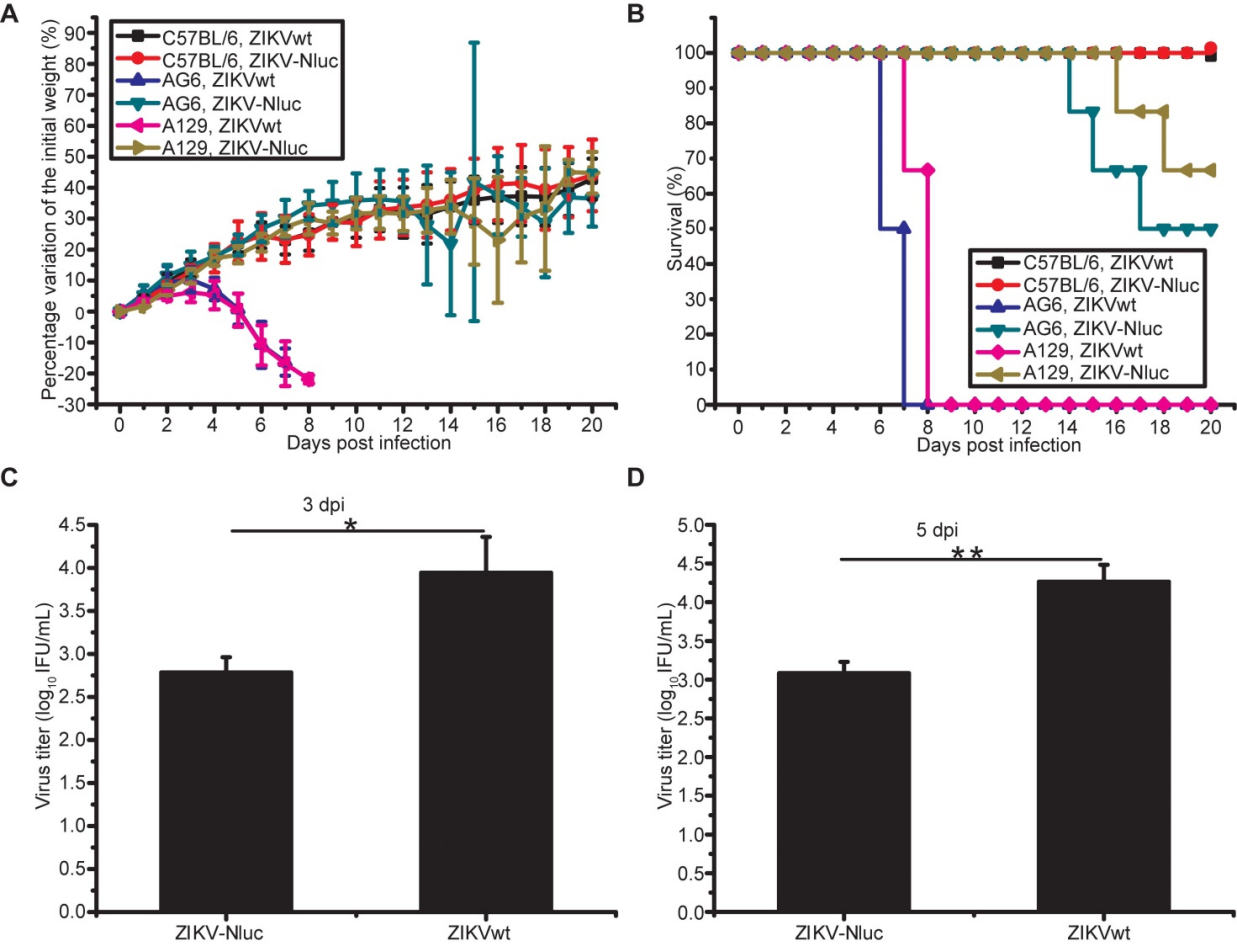
** Pathogenicity of ZIKV-Nluc in A129, AG6, and C57BL/6 mice.** (A, B) Groups of A129, AG6, and C57BL/6 mice (3-4 weeks old; n = 6) were infected intraperitoneally with 1.2 × 10^5^ IFU of WT or ZIKV-Nluc. Body weight loss and survival were monitored on a daily basis for 20 days. (C, D) Two groups of A129 mice (3-4 weeks old; n = 3) were infected intraperitoneally with 1 × 10^4^ IFU of WT or ZIKV-Nluc. Serum viral loads were determined at day 3 and day 5 by an immunostaining plaque assay. Data represent the mean ± SD analysed by Student's t-test (two tailed) (*, P < 0.05; **, P < 0.01; ***, P < 0.001).

To determine whether the ZIKV-Nluc virus causes a similar disease progression compared to ZIKVwt, mice were infected with 1.2 × 10^5^ IFU ZIKV-Nluc/ZIKVwt via the intraperitoneal (i.p.) route and monitored for 20 days for weight loss and mortality. Both A129 mice and AG6 mice infected with ZIKVwt showed weight loss starting at 4 dpi, and all mice died between 7 and 9 dpi (Figures 3A and B). By contrast, in the ZIKV-Nluc virus-infected mice, only 33.3% A129 mice and 50% AG6 showed significant weight loss and succumbed to infection by 19 and 18 dpi, respectively (Figures 3A and B). The immunocompetent mice, C57BL/6 mice, were not susceptible to infection with neither ZIKVwt nor ZIKV-Nluc (Figures 3A and B). The viral titres in sera obtained from A129 mice at 3 and 5 dpi were measured using an immunostaining focus assay (Figures 3C and D). A129 mice infected with ZIKV-Nluc were found to have significant different viral titres at both 3 dpi (P < 0.05) and 5 dpi (P < 0.01) compared to A129 mice infected with ZIKVwt (Figures 3C and D). These results showed that despite reduced levels of attenuation, the ZIKV-Nluc virus could develop detectable viral titres in sera, suggesting that the virus replicated well in immunodeficiency mouse models. In addition, C57BL/6 mice showed no clinical signs of disease or weight loss when infected with both ZIKV-Nluc and ZIKVwt.

### Using ZIKV-Nluc for the bioluminescence imaging of ZIKV infection

**Figure 4:**
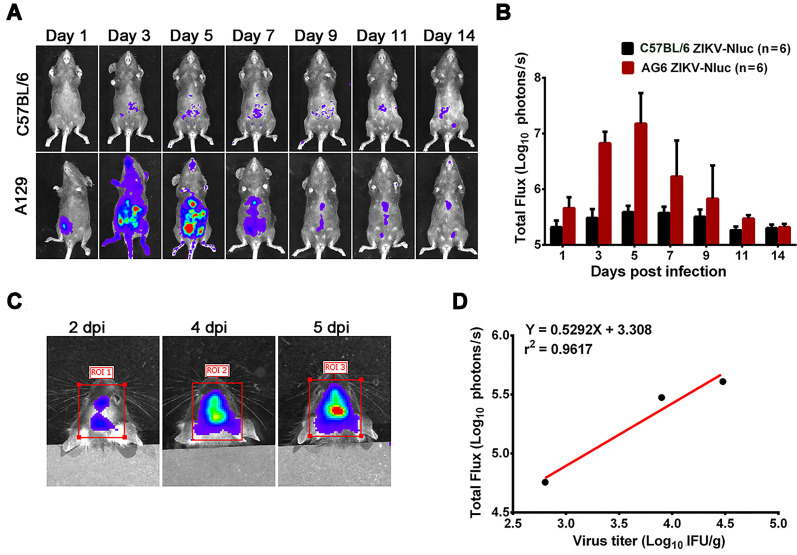
***In vivo* luminescence of ZIKV-Nluc-infected mice.** (A, B) Groups of A129 and C57BL/6 mice (3-4 weeks old; n = 6) were infected intraperitoneally with 1.2 × 10^5^ IFU of WT or ZIKV-Nluc. (A) Bioluminescence imaging of ZIKV-Nluc-infected mice was performed at the indicated times. Representative ventral views of the results were shown. (B) The average radiance of ZIKV-Nluc-infected mice was determined from region of interest (ROI) analysis of the ventral side. (C, D) Groups of AG6 mice (3-4 weeks old; n = 3) were infected with 6×10^3^ IFU of ZIKV-Nluc via the i.c. route. (C) Bioluminescence imaging of ROI from ZIKV-Nluc-infected mice was performed at the indicated times. (D) Linear correlation between the viral titres and Nluc signal values of the ZIKV-Nluc virus *in vivo*.

To investigate whether ZIKV-Nluc can be used as a tool for the bioluminescence imaging of ZIKV infection, A129 mice were inoculated with 1.2 × 10^5^ IFU ZIKV-Nluc via the i.p. route, and the bioluminescent signal was monitored at regular times post infection. As shown in Figures 4A and B, a rapid dissemination of bioluminescence from the injection site was observed in A129 mice as early as at 1 dpi, with the peak bioluminescence occurring at 5 dpi (a robust bioluminescence signal was detected in the whole abdomen, brain, limbs, and tail). For some C57BL/6 mice, a slight detectable luminance signal above background was observed at the indicated time points (Figures 4A and B). In a separate experiment, A129 mice were inoculated intracranially (i.c.) with 6 × 10^3^ IFU ZIKV-Nluc, and the bioluminescence in the brain regions were monitored at 2, 4, and 5 dpi. The titres of ZIKV-Nluc were measured using the double plaque assay. A direct correlation (r^2^ = 0.9617) was found between the viral titres of ZIKV-Nluc and the intensity of luminescence, indicating that the virus replication *in vivo* could be reflected by the changes in luminescence intensity (Figures 4C and D). To further validate the correlations between the bioluminescent signals and viral loads, AG6 mice were inoculated with 6 × 10^4^ IFU ZIKV-Nluc via the footpads. Tissues, including spleen, kidney, testis, and ileocecal junction, were isolated at 1, 3, and 5 dpi and subjected to bioluminescence imaging and viral load measurement. Linear regression analysis showed that Nluc signal values correlated well with viral RNA copies in mouse tissues (Figure S1). Collectively, using ZIKV-Nluc, the whole disease progression of the viral infection could be traced well via the IVIS CCD camera system.

### Involvement of type I and type II IFN in viral dissemination

**Figure 5:**
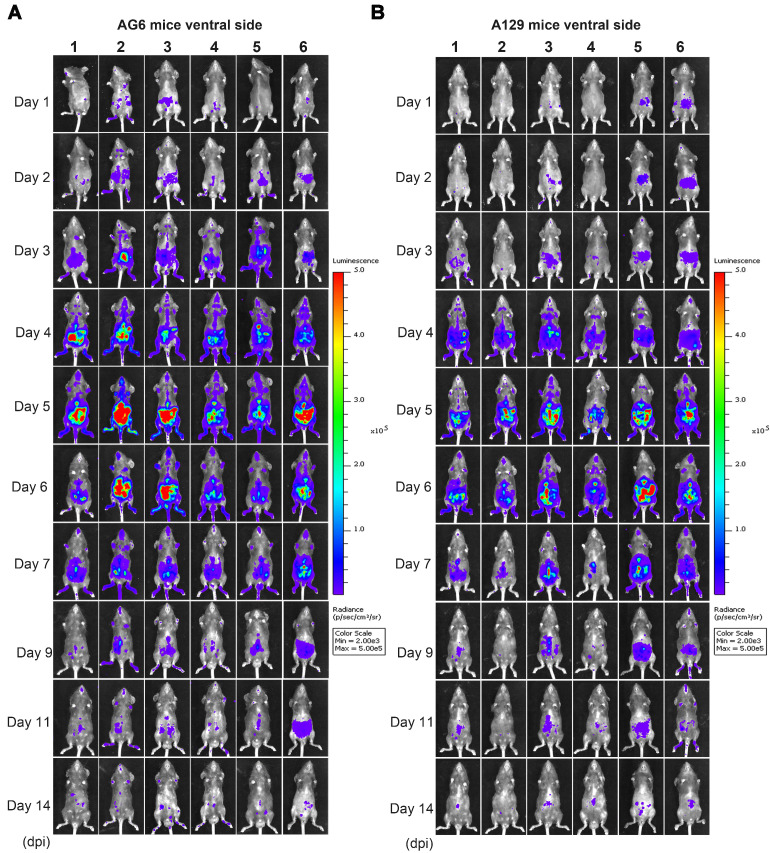
** Spatial and temporal progression of ZIKV-Nluc in AG6 and A129 mice in ventral views.** Groups of AG6 (A) and A129 (B) mice (3-4 weeks old; n = 6) were infected with 6 × 10^4^ IFU of WT or ZIKV-Nluc via the footpad. The viral spread of ZIKV-Nluc-infected mice was monitored in real time at the indicated times.

**Figure 6:**
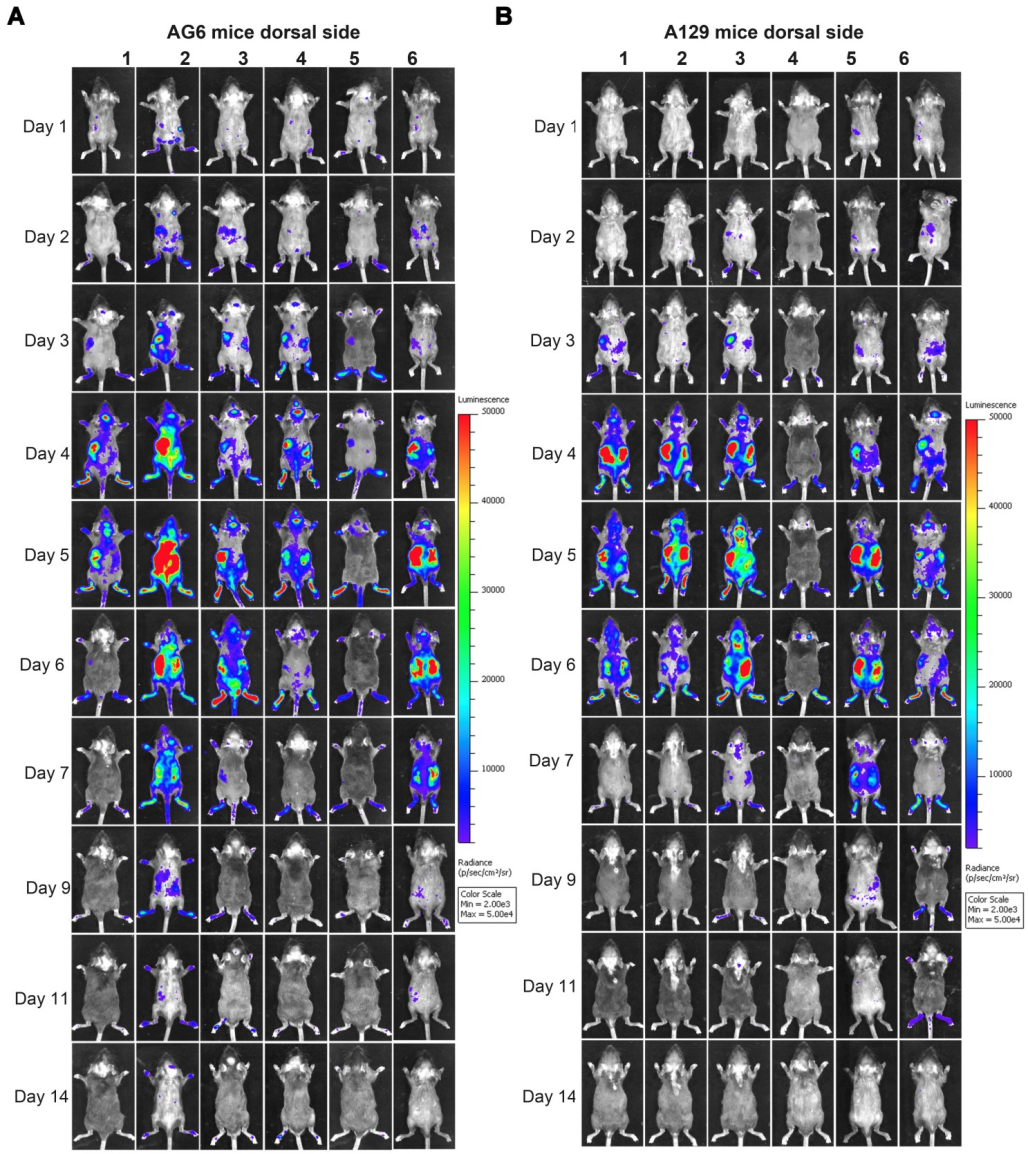
** Spatial and temporal progression of ZIKV-Nluc in AG6 and A129 mice in dorsal views.** Groups of AG6 (A) and A129 (B) mice (3-4 weeks old; n = 6) were infected with 6 × 10^4^ IFU of WT or ZIKV-Nluc via the footpad. The viral spread of ZIKV-Nluc-infected mice was monitored in real time at the indicated times.

To determine the mechanisms of ZIKV dissemination and pathogenesis in adults, pregnant mothers, and developing fetuses, the infection of mouse models by mimicking the natural infection route (footpad) is key 13, 28, 29. A129, AG6, and C57BL/6 mice were infected with 6 × 10^4^ IFU ZIKV-Nluc, or the parental ZIKV as control, via intra-footpad injections. The resulting bioluminescent signal was monitored longitudinally at regular time points post infection. No real or effective bioluminescence was detected in the mock-infected AG6, A129, and C57BL/6 mice, nor in mice infected with ZIKV-Nluc at 0 dpi (Figure S2). As shown in Figure 5 and Figure 6, in the infected A129 and AG6 mice, following the footpad injection, the bioluminescent signals were primarily observed at the local sites of inoculation and the peritoneal cavity at 1 dpi, further disseminating to the brain and other tissues or organs, peaking in the peritoneal cavity and brain at 5 dpi, and subsequently decreasing throughout the process of viral infection. These results revealed the complete process of ZIKV dissemination and showed that the ZIKV-Nluc virus primarily invaded various abdominal organs and the brain.

**Figure 7:**
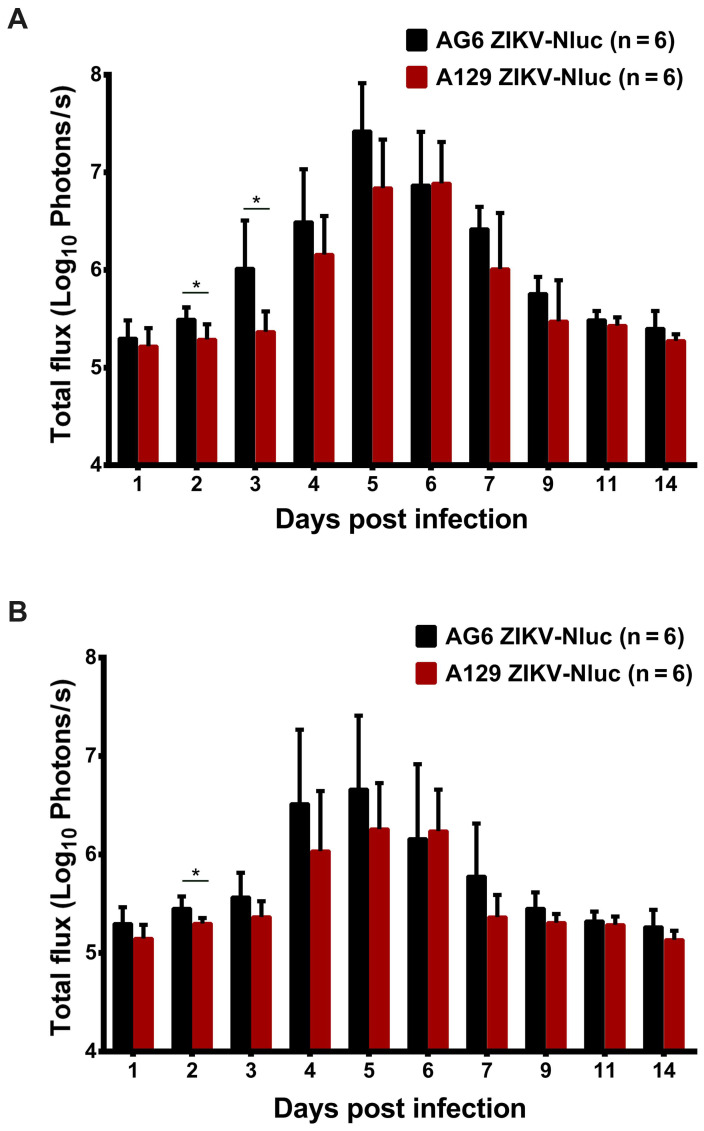
** Comparison of total fluxes from AG6 and A129 mice infected with ZIKV-Nluc.** (A) The average radiance of AG6 and A129 mice infected with ZIKV-Nluc (mice in Figure 5) was determined by the ROI analysis of the ventral side. (B) The average radiance of AG6 and A129 mice infected with ZIKV-Nluc (mice in Figure 6) was determined by the ROI analysis of the dorsal side. Data represent the mean ± SD analysed by Student's t-test (two tailed) (*, P < 0.05; **, P < 0.01; ***, P < 0.001).

At 9 dpi, only a slight bioluminescent signal was detected in the peritoneal cavity in the majority of the A129 and AG6 mice. However, at 14 dpi, the real and effective bioluminescence was still detectable in the peritoneal cavity of both AG6 and A129 mice (Figure 5 and Figure 6), highlighting the viscerotropism of ZIKV *in vivo*. By contrast, only a weak bioluminescent signal was detected in the peritoneal cavity of some C57BL/6 mice almost throughout the trial period. Next, we calculated the total flux of each ZIKV-Nluc-infected mouse, and found that the signal values of the AG6 mice were significantly higher than those of the A129 mice at 2 dpi for the dorsal side (P < 0.05), and at 2 and 3 dpi for the ventral side (P < 0.05) (Figures 7A and B). These results indicated that although type I IFNs were crucial for the viscera dissemination of ZIKV-Nluc, type II IFN also played a role in the process by delaying disease development in the early stages of virus infection.

### Tissue localization of ZIKV-Nluc

**Figure 8:**
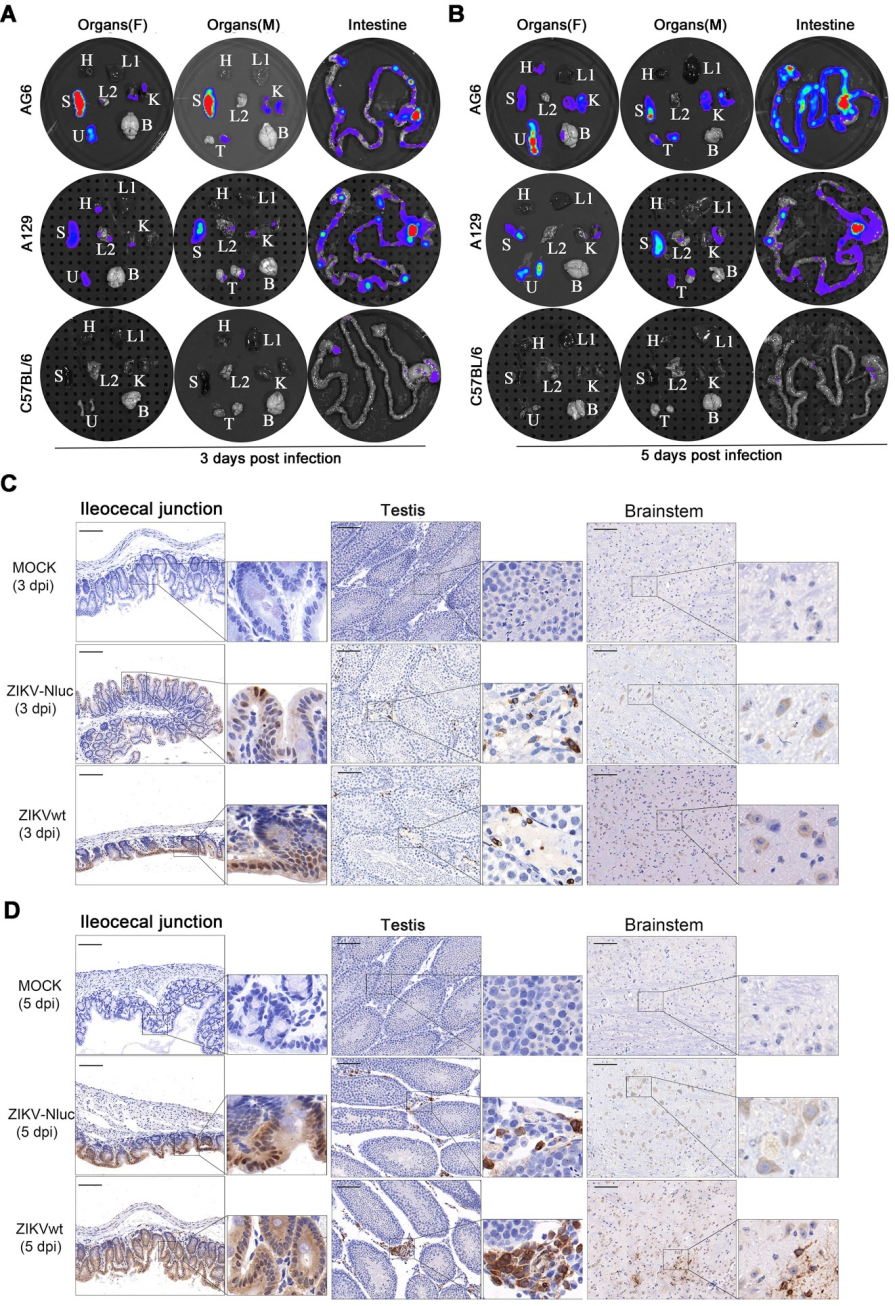
** Tissue localization of ZIKV-Nluc.** Groups of AG6, A129 and C57BL/6 mice (3-4 weeks old; n = 6) were infected with 6 ×10^4^ IFU of WT or ZIKV-Nluc via footpad. Immediately after bioluminescence imaging, ZIKV-Nluc-infected mice were sacrificed, and isolated organs including heart (H), liver (L1), spleen (S), lung (L2), kidney (K), uterus/ovary (U), testis (T), and brain (B) were subjected to *in vitro* bioluminescence imaging at 3 dpi (A) and 5 dpi (B). The expressions of E protein in ileocecal junction, testis and brainstem sections from infected AG6 mice were stained by immunohistochemistry at 3 dpi (C) and 5 dpi (D). Scale bars are 100 μm for lower magnification images (× 20) and boxes in lower magnification images indicated where the higher magnification images (× 80) were taken.

To accurately identify the potential target organs of ZIKV-Nluc in infected mice, the *in vitro* imaging of organs harvested from AG6, A129, and C57BL/6 mice infected with ZIKV-Nluc at 3 and 5 dpi was performed in a separate experiment. Marked Nluc signals were consistently observed in the abdominal organs of the infected AG6 and A129 mice, including in the spleen, intestine (i.e. ileocecal junction, a key region of the intestine), testis, uterus/ovary, and kidney at both time points (Figures 8A and B). Among these organs, the lymphoid rich organs, namely the spleen and the ileocecal junction, radiated two of the strongest light emissions, followed by the uterus/ovary, testis, and kidney. Bioluminescent signals in other organs, such as the brain, heart, and lung, varied individually among mice. For C57BL/6 mice, although only weak luciferase signals were detected in the intestines, no detectable bioluminescence was observed in other organs (Figures 8A and B). In addition, immunohistochemistry (IHC) staining of ileocecal junction, testis, and brain tissue sections showed a relatively clear distribution of E protein in AG6 mice infected with the ZIKV-Nluc virus (Figures 8C and D). By comparison, for C57BL/6 mice, the distribution of E protein was only found in ileocecal junction section at 5 dpi (Figure S3). Although the possibility that infection in other anatomical tissues may have occurred at earlier time points, or infected cells may have migrated from one location to another at later time points, these results supported previous observations that ZIKV preferentially replicated in both male and female reproductive tracts and led to infertility in mice 12, 30.

### Vertical transmission of ZIKV-Nluc

**Figure 9:**
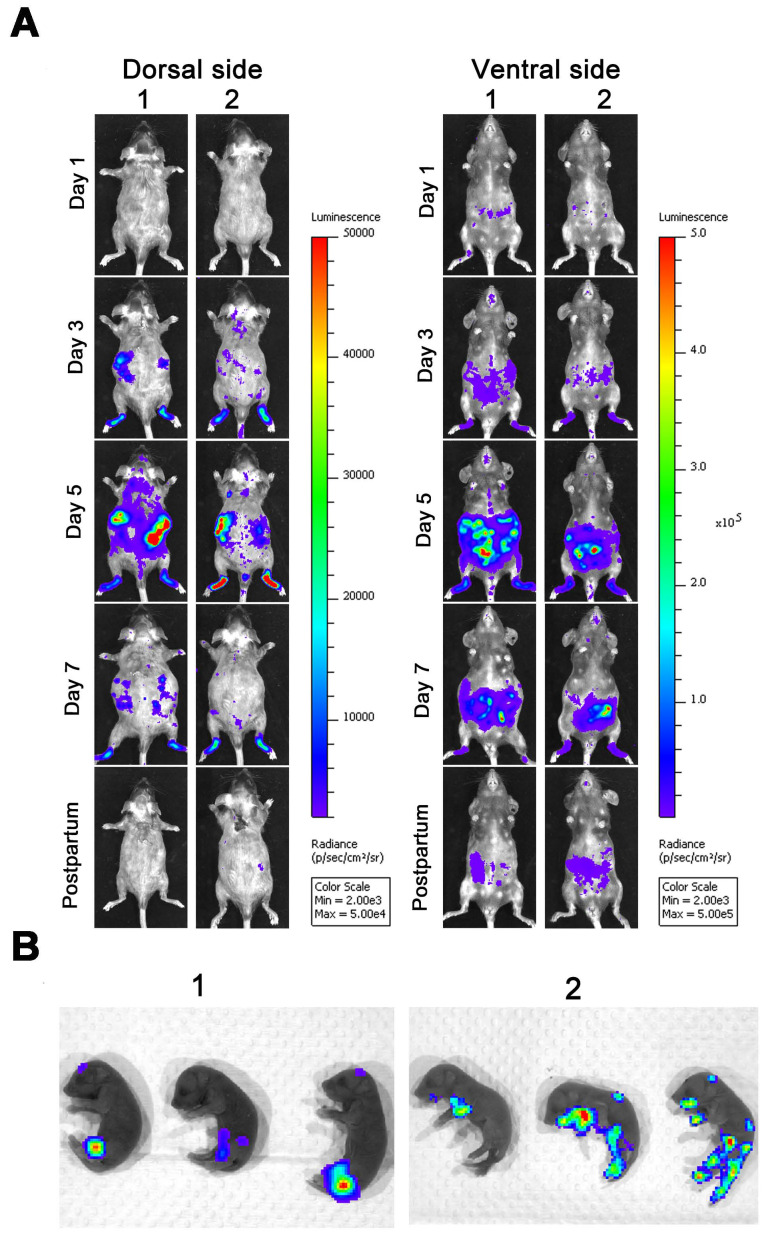
** Spatio-temporal dynamics of ZIKV-Nluc invading pregnant mice and spreading vertically to the fetuses.** Four-week-old AG6 female mice (n = 3) were mated with AG6 males. On E10, pregnant mice were infected with 6 × 10^4^ IFU of WT or ZIKV-Nluc via the footpad. Viral spreads from the ventral and dorsal views of ZIKV-Nluc-infected pregnant mice were monitored in real time at the indicated times (A). Bioluminescence imaging of fetuses from ZIKV-Nluc-infected dams was performed at day 1 after birth (B).

Given the fact that embryonic day 10-13 (E10-13, later in gestation) corresponds to the period of neurogenesis in mice 1, 31, 32, we infected pregnant dams with 6 × 10^4^ IFU ZIKV-Nluc at E10 via intra-footpad injection to determine the transmission of ZIKV from mother to offspring in mice. As shown in Figure 9A, in infected pregnant AG6 mice, the bioluminescent signals were primarily detected at the local site of inoculation and in the peritoneal cavity at 1 dpi, which then disseminated to other organs before peaking at 5 dpi, decreasing steadily until delivery. By comparison, only a slight bioluminescence signal was detected in the brains of infected pregnant AG6 mice at 3 and 5 dpi, which was markedly different from that of 3-week-old AG6 mice infected with ZIKV-Nluc. The peritoneal cavity of the maternal mice still radiated slight light emissions at 1 day postpartum. In terms of the newborn mice, 25 μl of diluted substrate via a single i.p. injection resulted in an exact and effective bioluminescence (Figure 9B), indicating that ZIKV was capable of crossing the maternal-fetal barrier to infect the fetuses through vertical transmission.

### Visualizing immunological protections of pooled immune serum

**Figure 10:**
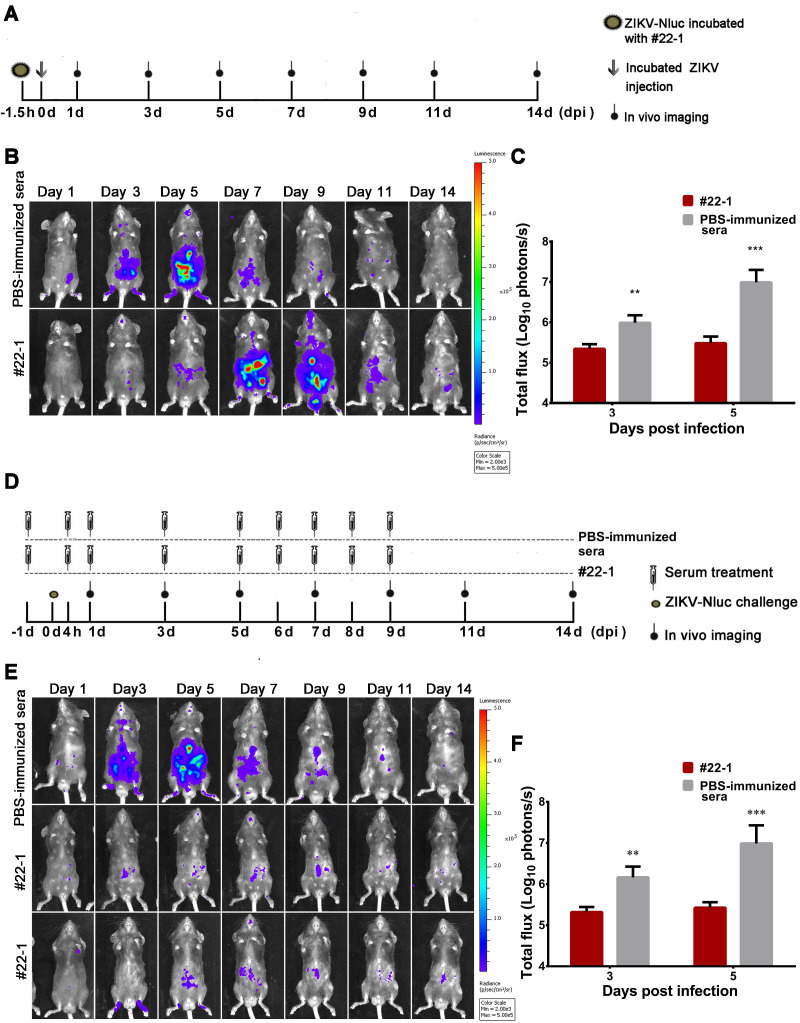
** Application of ZIKV-Nluc for immunological protection evaluation.** (A) Schematic representation of bioluminescence imaging of 3-4 weeks old AG6 mice receiving a mixture of ZIKV-Nluc and pooled immune serum, #22-1. (B) The viral spread from the ventral view of ZIKV-Nluc-infected mice was monitored in real time at the indicated times. (C) The average radiance of ZIKV-Nluc-infected mice was determined by ROI analysis of the ventral side. (D) Bioluminescence imaging of 3-4-week-old AG6 mice that received nine doses of #22-1. (E) The viral spread from the ventral view of ZIKV-Nluc-infected mice was monitored in real time at the indicated times. (F) The average radiance of ZIKV-Nluc-infected mice was determined by ROI analysis of the ventral side. Data represent the mean ± SD analysed by Student's t-test (two tailed) (*, P < 0.05; **, P < 0.01; ***, P < 0.001).

Although pooled immune sera (neutralizing anti-ZIKV antibodies) were recommended for protection against ZIKV infection 28, 33, it is unclear how these sera suppress the progression of viral infection *in vivo*. We adopted our established bioluminescence imaging to evaluate the immunological effect of a pooled immune serum, #22-1, which conferred robust neutralizing activities against ZIKVwt (NT_50_ titre of ~2048). Firstly, 80 IFU of the ZIKV-Nluc virus was pre-incubated with a 1:10 dilution of #22-1 at room temperature (RT) for 1.5 h before injection. The bioluminescent signal was monitored at regular time points post infection (Figure 10A). Pre-incubation with #22-1 significantly reduced the bioluminescence signal at 3 and 5 dpi (Figures 10B and C), and the bioluminescence of the #22-1-treated mice peaked in the peritoneal cavity and brain at 7 dpi (Figure 10B and Figure S4A). By contrast, the bioluminescence of the control mice peaked in the peritoneal cavity and brain at 5 dpi, before decreasing along with the process of viral infection (Figure 10B and Figure S4A).

Next, we designed a treatment regime of #22-1, as shown in Figure 10D. The pooled immune sera were administered at the indicated time points before and after infection with ZIKV-Nluc, while the bioluminescent signal was monitored at regular time points post infection. In the mice receiving PBS-immunized sera, a similar kinetic pattern of ZIKV-Nluc expression was observed in different tissues, with bioluminescence peaking at 5 dpi (Figure 10E and Figure S4B). Treatment with nine doses of #22-1 dramatically reduced the bioluminescent signal in almost all of the infected mice (Figures 10E and F, and Figure S4B). Taken together, the #22-1 treatment greatly restricted the spread of viral infection *in vivo*, which clearly demonstrated that the ZIKV-Nluc virus could be used as a tool for the real-time monitoring of how pooled immune sera (neutralizing anti-ZIKV antibodies or antiviral compounds) suppress the progression of viral infection.

## Discussion

ZIKV is a pathogenic virus that causes microcephaly, diffuse calcifications, GBS, meningoencephalitis, and other neurological complications in humans 4, 8, 34. In this study, we generated a replication-competent Nluc reporter ZIKV, which is genetically stable *in vitro* and* in vivo*, which represents a powerful tool for the monitoring of the spatio-temporal dynamics of viral infection in living mice. For the first time, we report on the complete process of ZIKV dissemination, as well as the identification of the ileocecal junction as a crucial visceral target of viral infection, the tracking of the vertical propagation of ZIKV, and the congenital infection of fetuses during pregnancy. Our findings confirmed the utility of the reporter virus for use in immunological protection or therapeutic efficacy studies using mouse models.

In the generated ZIKV-Nluc virus, the Nluc reporter was expressed as an additional part of the viral polyprotein, followed by its cleavage from the capsid protein mediated by 2A protease. Notably, C38, which retained the 5' sequence downstream of the AUG region (5'DAR), the hairpin in the C protein-coding region (cHP), the 5'cyclization sequence (5'CS), and the sequence downstream of the 5'CS-pseudoknot (DCS-PK) 35-37, was duplicated upstream of the reporter gene to ensure viral RNA replication. Similar strategies have been applied in previous studies to generate several flaviviruses carrying reporter genes 27, 38-41. A major hurdle in the practical application of these reporter flaviviruses is the relative instability of viral genomes. It has been proposed that engineered viruses evolve during their lifetime, and that a stable reporter virus could be harvested by picking small plaques 27. In our study, a strain of P8 ZIKV-Nluc virus was found to be stable after five rounds of viral infection with no nucleotide substitution in the genome. We postulated that the high stability of P8 ZIKV-Nluc was due to the very small Nluc gene (513bp), the P0 stocks as a population (quasispecies) of general consensus sequence might contain few viral particles with mutant genomes, after purification, the single virus yields exhibited homogeneous entity and high genetic stability. Further studies are still warranted to investigate the reason for the high stability of ZIKV-Nluc. Heterologous gene insertions normally result in the attenuation of the constructed viruses both *in vitro* and *in vivo* 17, 19, 24, 41-43. As expected, the purified ZIKV-Nluc exhibited a lower replication kinetic in the cell culture and a relatively low pathogenicity in mice. Despite this, the ZIKV-Nluc virus showed a similar growth pattern compared to that of its parental counterpart and produced robust luciferase activities with a peak value of > 2 × 10^8^ light units. The magnitude of the bioluminescence generated by ZIKV-Nluc correlated well with its titre in both the cell culture and mice, which was in agreement with the findings reported by studies on JEV and DENV reporter viruses 19, 24. Such properties of ZIKV-Nluc provide a powerful means for further characterizing ZIKV dissemination in living mice using bioluminescence imaging.

Using the ZIKV-Nluc virus, we visualized the real-time ZIKV infection in A129, AG6, and C57BL/6 mice. ZIKV is known to directly infect neuronal progenitor cells and causes microcephaly, among other severe pathological complications 44-46. Here, we demonstrated that ZIKV-Nluc primarily spread to the peritoneal cavity in A129, AG6, and C57BL/6 mice at 1 dpi, and was sustained throughout the remaining infection course. We hypothesized that the high viral loads in the peritoneal cavity may contribute to the quick dissemination of ZIKV to the brain and other organs. Although ZIKV-Nluc was found to be confined to specific parts of the peritoneal cavity in immunocompetent C57BL/6 mice, the lack of type I IFN receptors in A129 mice is likely to have contributed to the rapid proliferation of the virus, as well as to its spread to the brain and other organs, which was consistent with a previous biochemical analysis that found that ZIKV did not antagonize type I IFN response by promoting the degradation of STAT2 and did not induce disease in immunocompetent mice 47. By studying genetically deficient animals, previous studies have demonstrated that type I IFNs are crucial for the dissemination of JEV to visceral organs 19. Type I IFNs serve as one of the key components in the innate immune system, and many interferon stimulated genes were found to be essential for viral restriction and clearance 48, 49. After infection with a neurotropic virus, such as West Nile virus, the induction of type I IFN expression in the endothelium has been found to enhance tight junction integrity and limit the permeability of the blood‒brain barrier 17, 48, 50. We also investigated the function of type II IFNs in the control of ZIKV infection. Compared with the singly-deficient A129 mice, AG6 mice lacking both type I and type II IFN receptors were found to be more susceptible to ZIKV-Nluc, with significantly higher bioluminescent signals being observed at 2 dpi for the dorsal side and at 2 and 3 dpi for the ventral side, indicating that type II IFNs contribute to limiting systemic ZIKV infection in mice in the early stage. Neurons in the central nervous system (CNS) have a limited regeneration ability, implying that the noncytolytic clearance of virus from neuronal cells, rather than direct neuronal lysis, is required to maximize the preservation of CNS function 51. Type II IFNs were previously found to non-cytolytically clear Sindbis virus and measles virus from infected CNS neurons 52, 53. However, the protection of the noncytolytic immune response against neurotropic flavivirus has been disputed 51, 54. Given that type II IFNs interact with type I IFNs through distinct as well as common IFN receptor complexes 55, we proposed that both type I and type II IFNs are functionally non-redundant for the anti-ZIKV defence, and may modulate virus dissemination by restricting infection in extraneural tissues before irreversible CNS damage in mice. Further studies will be needed to determine the exact mechanism of the non-cytolytic clearance of ZIKV from infected neurons in the CNS.

ZIKV is known to preferentially replicate in the reproductive tract, including in the testis, uterus, and ovary 12, 30, and this has been was confirmed by the bioluminescence imaging of specific organs. However, whether the intestine is a potential target organ of ZIKV infection remains to be further clarified. In this study, we found that the Nluc signal was widespread in the whole intestine in all of the immunodeficient mice tested, indicating that the intestine is likely to serve as an important organ of ZIKV infection. Previously, luciferase signals were also detected in the intestine of singly-deficient A129 mice infected with JEV-Rluc or doubly-deficient AG129 mice infected with DENV-Fluc, suggesting that JEV and DENV can replicate in gut-associated lymphoid tissues 19, 24. Nevertheless, it remains unclear which intestine segment is the exact ROI prone to viral infection. In this study, we excised the whole intestine of infected mice, and found that the anatomical location radiating the strongest light emission was the ileocecal junction. The ileocecal junction functions as a mechanical barrier against colonic reflux, and is involved in the “ileal brake”, which slows the transit of chyme through the intestinal tract 56, 57. The fact that ZIKV targets the ileocecal junction is consistent with a recent study that found that the infection of enteric neurons with neurotropic flaviviruses causes delayed gastrointestinal transit in mice 58.

Our study elucidated the spatio-temporal dynamics of ZIKV in pregnant mice and its subsequent vertical spread to the fetuses. The placenta develops within days of conception and acts as an innate barrier to invading microorganisms 59, 60. Based on epidemiological data combined with the detection of proteins and nucleic acids, it has been previously suggested that ZIKV can cross the placental barrier, being directly associated with fetal death, microcephaly, and other fetal abnormalities during pregnancy 1, 61. However, the mechanism for the infection and dissemination of ZIKV at the different stages of gestation remains unknown. In this study, by infecting pregnant AG6 mice with ZIKV-Nluc, the virus was found to primarily target the local site of inoculation and peritoneal cavity, subsequently spreading to other organs. The fetus may be highly sensitive to ZIKV infection during the first trimester of pregnancy 62. However, devastating fetal outcomes, such as microcephaly, cerebral atrophy, ventricular enlargement, and cerebral calcifications, have also been found at other various gestational ages 59, 61. Our results clearly demonstrated that the virus crossed the maternal-fetal barrier and infected newborn pups when the pregnant AG6 mice were infected at E10, corresponding to the period of neurogenesis in mice 1, 31. Further longitudinal studies are needed in order to define the relationship between the severity of maternal infection and fetal consequences, as well as to determine the mechanism by which ZIKV crosses the placental barrier at the different stages of gestation.

We used the Nluc-expressing virus to visualize the neutralizing activities and therapeutic potential of the immune serum #22-1 in mice. As expected, significant reductions in bioluminescence signal were observed in mice receiving a pre-incubated virus-immune serum mixture in the early stages of infection. The bioluminescence in mice peaked at 7 dpi, indicating that pre-incubation before inoculation may not guarantee the neutralization of all the infectious particles. Nevertheless, these results provide new evidence for the superior genetic stability of ZIKV-Nluc* in vivo* compared with other reporter flaviviruses 19, 24. As a proof-of-principle experiment, we traced the infection and clearance of ZIKV-Nluc in A129 mice treated with several doses of #22-1. The imaging results showed that a significant reduction of bioluminescence was observed in the mice that received a 9-dose treatment of antiviral serum, indicating that #22-1 exhibits therapeutic activity similar to that of other reported antibodies 33, 63. Previously, bioluminescence imaging has been recommended to predict lethality and evaluate the efficacy of vaccines and therapeutic strategies in mice 64. Here, we demonstrated that the ZIKV-Nluc virus may provide a new means for the development of antiviral therapeutics and the preclinical evaluation of vaccines.

Despite its merits, this study also contains some limitations. One limitation was that ZIKV-Nluc exhibited a lower replication efficiency and a lower pathogenicity than ZIKVwt. Of note, heterologous gene insertions always led to the attenuation of recombinant RNA viruses 17, 19, 24, 41-43. Although ZIKV-Nluc was used to determine the replication, dissemination, and pathogenesis of ZIKV, as well as evaluate antivirals and vaccines, further studies will be needed to address the issue of attenuation. The second limitation of this study was the immunocompromised mice that were used. A129 and AG6 mice have been used previously to mimic aspects of ZIKV infection in humans. However, due to the lack of key components of antiviral immunity, these mouse models may not reveal the full range of disease manifestations in humans 11, 65. Immunologically competent mice treated with IFNAR1-blocking monoclonal antibody 65 or humanized mouse models may achieve better results in future studies.

In summary, a novel ZIKV reporter virus was established for the *in vivo* imaging of ZIKV. This study is the first to investigate the spatio-temporal dynamics of ZIKV replication, the invasion of immune-sheltered tissues by ZIKV, and the vertical propagating of ZIKV during pregnancy. In addition to the brain and reproductive tract, including the testis, uterus, and ovary, the intestine was also demonstrated to be a potential target of ZIKV dissemination, wherein the ileocecal junction may likely play a key role in the neuronal dysfunction of ZIKV infection. The non-invasive imaging of ZIKV-Nluc is a powerful tool for use in the characterisation of the replication, dissemination, and pathogenesis of ZIKV, as well as for the evaluation of antivirals and vaccines *in vivo* for the treatment of ZIKV infection in humans.

## Materials and Methods

### Ethics statement

All animal experiments were conducted in strict accordance with the institutional guidelines for animal research and approved by the Administration of Affairs Concerning Experimental Animals of the People's Republic of China. All animal treatments were reviewed and approved in advance by the Ethics Committee of the Animal House facility of Wuhan Institute of Virology, Chinese Academy of Sciences (permit no. WIVA07201603).

### Cells and viruses

African green monkey kidney epithelial cells (Vero; CCL-81; ATCC) were cultured in Dulbecco's modified Eagle's medium (DMEM) (Invitrogen, Darmstadt, Germany) containing 10% fetal bovine serum (FBS) (Life Technology, Australia), 100 U/mL penicillin, and 100 μg/mL streptomycin maintained in 5% CO_2_ at 37 °C. The parental ZIKV was rescued by transfecting Vero cells with the full-length cDNA clone, ZIKV-FL, as previously described 10.

### Plasmid construction and DNA transfection

To generate the infectious clone of ZIKV-Nluc, ZIKV-FL was used as a backbone to insert the Nluc reporter gene. As shown in Figure 1A, fragment 1, covering “CMV promoter-5'UTR-C38”, and fragment 3, covering “C-prM-E187”, were amplified using ZIKV-FL as a template. Fragment 2, covering (Nluc-2A), was amplified using the pNL1.1 [Nluc] vector (Promega, Madison, USA) as a template. Fragments 1-3 were fused together and cloned into ZIKV-FL at the *Kpn* I and *Avr* II sites, yielding ZIKV-Nluc-FL. Before transfection, ZIKV-Nluc-FL was verified by restriction enzyme digestion and complete sequencing.

The full-length cDNA clone ZIKV-Nluc-FL under the control of the CMV promoter was used to produce infectious viruses. The transfection was performed as previously described 10. In brief, Vero cells at 80% confluence in 35 mm culture dishes were transfected with ZIKV-Nluc-FL by lipofectamine 3000 (Life Technologies). The supernatant was harvested at 3 days post transfection (dpt), clarified by centrifugation, and stored at ‒80 °C.

### Immunofluorescence assay (IFA)

The cells infected with ZIKVwt or ZIKV-Nluc were washed once with cold phosphate-buffered saline (PBS) and fixed with cold (‒20 °C) methanol-acetone (1:1) fixation solution for 12 min at RT. The fixed cells were washed with PBS three times and incubated with an anti-ZIKV envelope (E) protein MAb (BioFront Technologies, FL, USA) (diluted 1:200) for 1 h. After washing, the cells were incubated with goat anti-mouse IgG conjugated to FITC (Proteintech, Wuhan, China) (diluted 1:200) at 37 °C for 60 min. After washing again, the cell nuclei were dyed with Hoechst 33258 at 37 °C for 10 min. The images were photographed with a NIKON fluorescence microscope (Tokyo, Japan).

### Plaque assay and immunostaining focus assay

For the double plaque assay, Vero cells at 80% confluence in 6-well plates were inoculated with 500 μl of 10-fold serial dilutions of viral samples in serum-free DMEM. After 1.5 h incubation, 3 mL of 0.6% agarose supplemented with 2% FBS was added into each well. After incubation for 4 days, 3 mL of agarose containing 0.33% neutral red was added to each well. The plaques were photographed or picked after incubation for another 24 h.

The immunostaining focus assays were performed as previously described 10. In brief, Vero cells in 24-well plates were inoculated with 100 μl of 10-fold serial dilutions of viral samples for 1.5 h. Then, 1 mL of 1.25% methyl cellulose overlay was added to each well and the cells were incubated at 37 °C for 4 days. Cells were fixed in methanol-acetone fixation solution. After washing three times, the cells were incubated with ZIKV-specific hyperimmune mouse serum. After washing three more times, the cells were incubated with HRP-conjugated second antibodies. Finally, the viral foci were visualized by addition of the DAB (3, 3-diaminobenzidine) HRP substrate using an Enhanced HRP-DAB kit (Tiangen, China), according to the manufacturer's instructions.

### Luciferase assay

Vero cells at 80% confluence in 24-well plates were infected with the ZIKV-Nluc virus. After incubation in 5% CO_2_ at 37 °C, the cells were lysed with passive lysis buffer at the indicated time points. Luciferase activities were measured by using the Nano-Glo^®^ Luciferase Assay System (Promega), according to the manufacturer's instructions.

### Animal experiments

The strains of mice used in this study were A129 (IFN-α/β receptor deficient), AG6 (IFN-α/β and IFN-γ receptor deficient), and C57BL/6. A129 and AG6 mice were kindly provided by Gengfu Xiao (Wuhan Institute of Virology, Chinese Academy of Sciences) and Qibin Leng (Institute Pasteur of Shanghai, Chinese Academy of Sciences), respectively. C57BL/6 mice were purchased from HuBei Center for Disease Control (CDC) (Wuhan, China). All mice were bred under specific pathogen-free conditions in the Animal Resource Center at the Wuhan Institute of Virology, Chinese Academy of Sciences.

Mice were infected with 1.2 × 10^5^ IFU ZIKV-Nluc/ZIKVwt by intraperitoneal (i.p.) injection, or 6 ×10^4^ IFU ZIKV-Nluc/ZIKVwt by footpad injection. PBS was injected into the mock-infected mice by the same route. The clinical course of viral infection was monitored by survival, weight loss, and disease symptoms.

For tissue localization analysis, 3-4-week-old mice that had received footpad injections were anesthetized, imaged, and subjected to tissue collection (heart, liver, spleen, lung, kidney, brain, testes, ovary/uterus, and intestine). All of the tissues were imaged *in vitro*, then stored at ‒80 °C for later use. For the study of vertical transmission, pregnant mice were infected at embryo day 10 (E10) by footpad injection and subjected to imaging *in vivo* at the indicated time points. The newborn mice were also examined by bioluminescence imaging at 1 day after birth.

### Titration of virus from excised tissues

For the measurement of the viral titre, 3-4-week-old AG6 mice that that had received i.c. injections were anesthetized, imaged, and subjected to tissue collection. At 2, 4, and 5 dpi, the brains of the infected mice were removed, weighed, and homogenized with zirconia beads in 1 mL of DMEM. Then, the viral titres in the brains were quantified using immunostaining focus assays.

For the measurement of viral loads, 3-4-week-old AG6 mice that had received footpad injections were anesthetized, imaged, and subjected to tissue collection. At 1, 3, and 5 dpi, the spleen, kidney, testis, and ileocecal junction of the infected mice were removed, weighed, and homogenized with zirconia beads in 1 mL of TRIzol reagent. Next, the viral loads in tissues was quantified using qRT-PCR, as described previously 10. Briefly, the total RNA was extracted from various tissues using TRIzol reagent before being reverse transcribed into cDNA by using the PrimeScript RT reagent kit. A pair of primers (ZIKV-F: AARTACACATACCARAACAAAGTG and ZIKV-R: TCCRCTCCCYCTYTGGTCTTG) 66 was used to amplify a conserved sequence of the NS5 gene. The cycling programme comprised 95 °C for 3 min, 40 cycles of 95 °C for 10 s, 55 °C for 10 s, and 65 °C for 45 s.

### Bioluminescence imaging

To perform the bioluminescence imaging, the infected mice were shaved in advance and anaesthetised via the subcutaneous (s.c.) injection of Avertin (150 μl/10 g of 2.5% solution). The Nano-Glo substrate (Promega) was diluted 1:20 in PBS, and each mouse was i.p. injected with 100 μl of the mixture. The bioluminescence data were collected using an IVIS CCD camera system (Caliper Life Science), and further processed in Living Image (version 4.5) software (Caliper Life Sciences). To analyse the bioluminescence signals, the ROIs were selected manually in the uniformly scaled images, and the data were defined as total flux in photons/second.

### Nano-Glo luminescence-based ZIKV neutralization assay

A Nano-Glo luminescence-based ZIKV neutralization assay was developed for the measurement of the ZIKV-specific neutralizing antibodies, according to a previously described assay that used plaques as a measurement 10. Briefly, the serum samples were two-fold serially diluted, and incubated with 80 IFU of the ZIKV-Nluc virus at 37 °C for 1.5 h. Then, 100 μl of virus-serum mixture was added to Vero cells at 80% confluence in 24-well plates. After incubation at 37 °C for 1.5 h, the cells were washed once with PBS, and were then cultured in freshly prepared medium containing 2% FBS. At 48 hours post infection (hpi), the levels of luciferase activity were measured using the Nano-Glo^®^ Luciferase Assay System. The 50% neutralization titre (NT_50_) was defined as the reciprocal of the highest dilution of each serum sample that resulted in a 50% reduction of the relative light unit relative to the control samples. The traditional neutralization method, PRNT_50_, was also performed to ensure the accuracy of the Nano-Glo assay.

### Immunohistochemistry

The immunohistochemistry assays were performed as previously described 5, 10. In brief, the tissues were fixed, embedded in paraffin, sectioned at a thickness of 5 μm, and mounted onto slides. After deparaffinization and antigen retrieval, the sections were incubated overnight at 4 °C. After washing three times, the sections were incubated with HRP-conjugated second antibodies and visualized using DAB reagent (Envision system kit; Dako). The slides were counterstained with haematoxylin and eosin. Images were captured using a whole-slide digital Pannoramic scanner (3D-Histech, Budapest, Hungary).

### Statistical analysis

Student's t-test and analysis of variance (ANOVA) were used to analyse all of the virologic and immunologic data for significant differences (p < 0.05). The statistical analyses were performed in IBM SPSS Statistics v18.0 (Chicago, IL, USA).

## Supplementary Material

Supplementary figures and tables.

Fasta data-S1.

## Abbreviations

ZIKV: zika virus
GBS: Guillain-Barre syndrome
Fluc: firefly luciferase
Rluc: Renilla luciferase
Nluc: nanoluciferase
FMDV2A: foot and mouth disease virus 2A sequence
CCD: charge-coupled-device
NPCs: neuronal progenitor cell
WNV: West Nile virus
CNS: central nervous system
ROI: region of interest
