# Supplementary material for: In vivo imaging of Zika virus reveals dynamics of viral invasion in immune-sheltered tissues and vertical propagation during pregnancy
Source: Theranostics. 2020 May 16;10(14):6430–47. doi: 10.7150/thno.43177 (PMC7255039; doi:10.7150/thno.43177)
Supplement: Supplementary file 1 — Supplementary figures and tables. [file thnov10p6430s1.pdf]

Figure S1

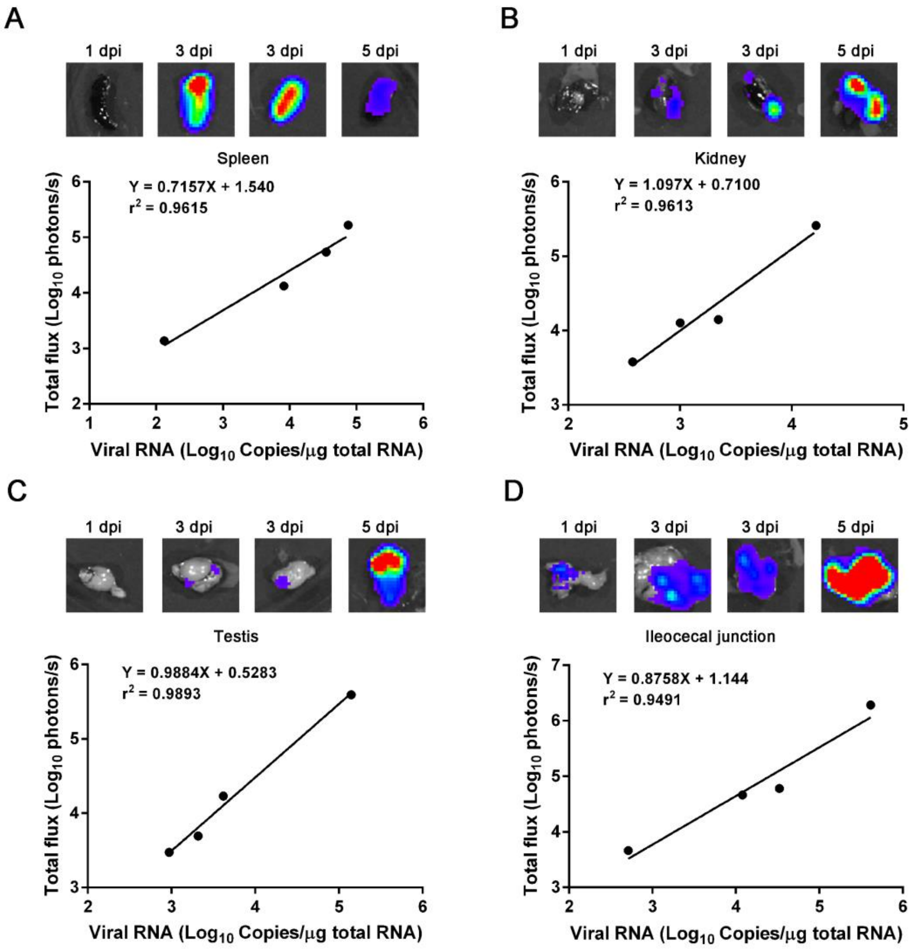

Figure S1. Linear correlations between viral loads of and Nluc signal values. Groups of AG6 mice (3-4 weeks old; n = 3) were infected with 6 $\times 10^4$ IFU of ZIKV-Nluc via footpad. Immediately after bioluminescence imaging, the mice were sacrificed, and tissues including spleen (A), kidney (B), testis (C), and ileocecal junction (D) were isolated and subjected to bioluminescence imaging and viral load measurement by qRT-PCR at the indicated times. Linear correlations between viral RNA copies and Nluc signal values in mouse tissues were determined.

Figure S2

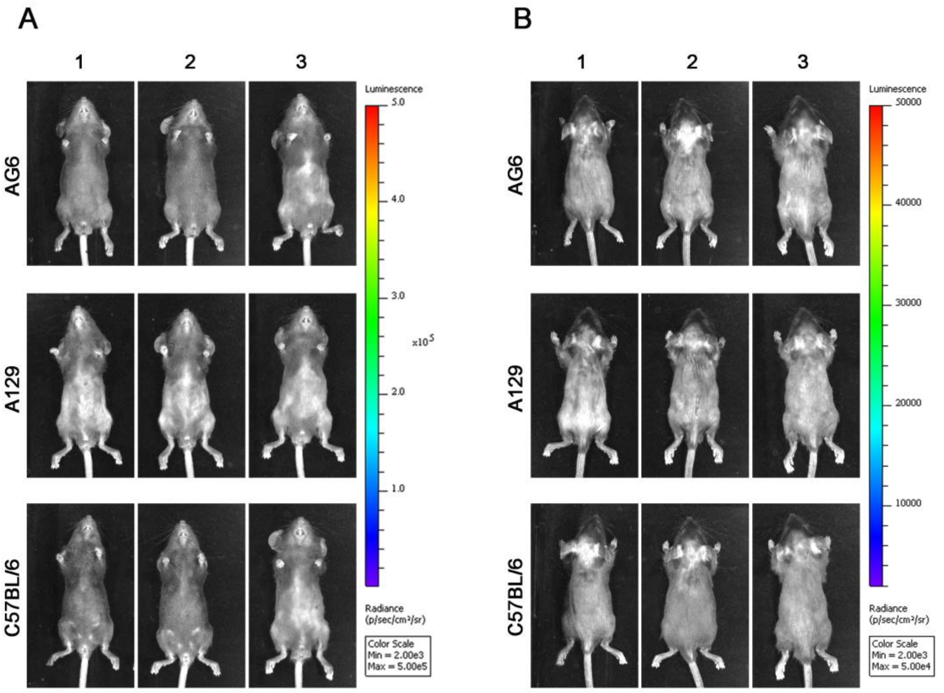

Figure S2. *In vivo* bioluminescence of AG6, A129 and C57BL/6 mice infected with ZIKV-Nluc. Groups of mice (3-4 weeks old; n = 3) were infected with 6×10^4 IFU of ZIKV-Nluc. Bioluminescence imaging of the mice in ventral view (A) and dorsal view (B) was performed at 0 dpi.

Figure 3

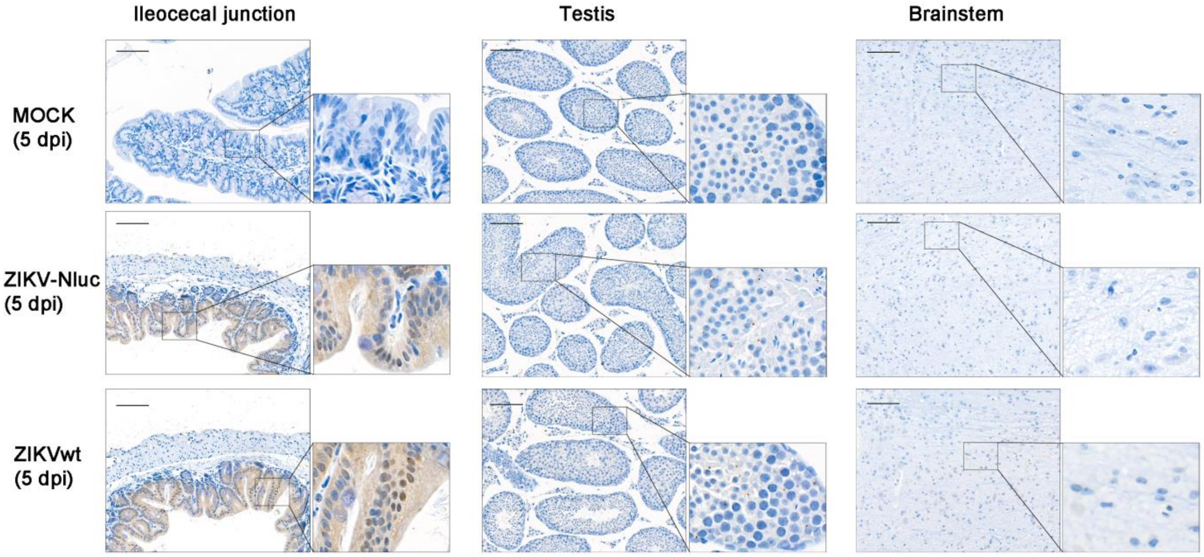

Figure S3. Tissue localization of ZIKV-Nluc. Groups of C57BL/6 mice (3-4 weeks old; n = 6) were infected with 6 $\times 10^4$ IFU of WT or ZIKV-Nluc via footpad. Immediately after bioluminescence imaging, ZIKV-Nluc-infected mice were sacrificed at 5dpi. Ileocecal junction, testis and brain were excised and the expressions of E protein in these tissue sections were stained by immunohistochemistry (scale bar = 100 μm).

Figure S4

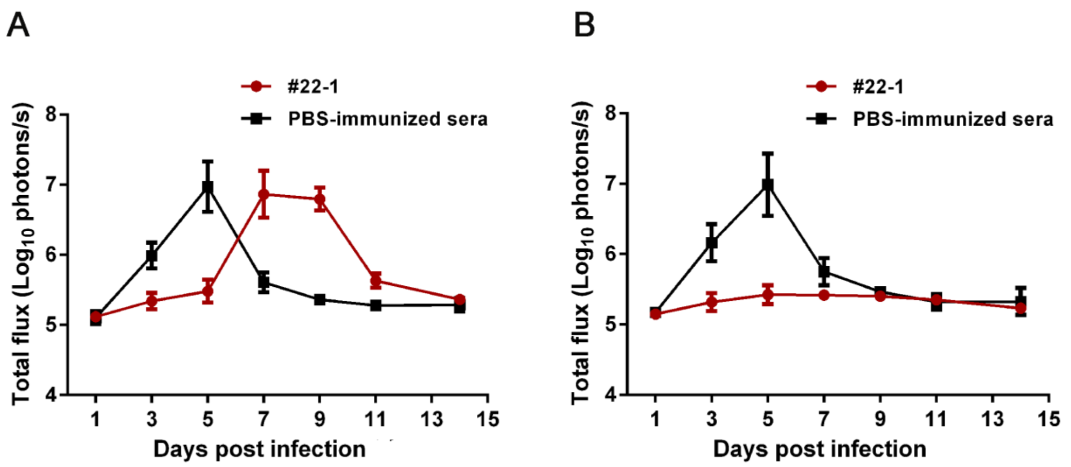

Figure S4. Total fluxes of AG6 mice infected with ZIKV-Nluc. (A) The average radiance of AG6 infected by the mixture of ZIKV-Nluc and #22-1 (mice in Figure 10B) was determined from ROI analysis of the ventral side at all the indicated times. (B) The average radiance of AG6 infected by ZIKV-Nluc (mice in Figure 10E) was determined from ROI analysis of the ventral side at all the indicated times.
